# Supplementary material for: Stroke volume and cardiac output during 6 minute-walk tests are strong predictors of maximal oxygen uptake in people after stroke
Source: PLoS One. 2022 Aug 30;17(8):e0273794. doi: 10.1371/journal.pone.0273794 (PMC9426911; doi:10.1371/journal.pone.0273794)
Supplement: Attachment — Submitted filename: Letter to the editor August 2022.docx [file pone.0273794.s003.docx]

August 2022

Dear Editorial Manager at PLOS ONE,

Regarding: “Stroke volume and cardiac output during 6 minute-walk tests are strong predictors of maximal oxygen uptake in people after stroke”

Thank you for including me in this process and for having the confidence in letting me review your interesting work. Comments and suggestions for improvement have been met and made and I recommend the article to be published.

**Henriette Busk**

Assistant Professor

Cand. Scient. Fys, PhD.

**Københavns Universitet**

Department of Geovidenscience and Natural Ressource management

Rolighedsvej 23

2000 Frederiksberg

DIR 23250898

[hebu@ign.ku.dk](mailto:hebu@ign.ku.dk)
